# Transthoracic Echocardiography-Assisted, Low-Fluoroscopy Left Bundle Branch Area Pacing: A Comparison of Implantation Approaches and Lead Types

**DOI:** 10.3390/diagnostics16193172

**Published:** 2026-09-29

**Authors:** Po-Hua Chu, Chung-Lieh Hung, Kuo-Tzu Sung, Shu-Fen Hsu, Cheng-Ting Tsai, Jen-Yuan Kuo, Sheng-Hsiung Chang

**Affiliations:** 1 Cardiovascular Division, Department of Internal Medicine, MacKay Memorial Hospital, Taipei 104217, Taiwan; 2 Department of Nursing, MacKay Junior College of Medicine, Nursing, and Management, New Taipei City 252005, Taiwan; 3 Institute of Biomedical Sciences, MacKay Medical University, New Taipei City 252005, Taiwan; 4 Department of Medicine, MacKay Medical University, New Taipei City 252005, Taiwan; 5 Department of Telehealth, MacKay Memorial Hospital, Taipei 104217, Taiwan; 6 School of Nursing, MacKay Medical University, Taipei 252005, Taiwan

**Keywords:** transthoracic echocardiography, left bundle branch area pacing, fluoroscopy time, lumen-less pacing lead, stylet-driven lead

## Abstract

**Background:** Transthoracic echocardiography (TTE)-assisted left bundle branch area pacing (LBBAP) is a potential supplementary to fluoroscopy-guided implantation and may reduce radiation. **Objectives:** To evaluate the feasibility and long-term stability of TTE-assisted, low-fluoroscopy LBBAP across different workflows and lead types. **Methods:** This retrospective study enrolled 45 consecutive patients between November 2021 and June 2022, analyzing 42 successful cases. Patients were stratified by workflow approach (non-scrubbed, *n* = 24; scrubbed aseptic, *n* = 18) and lead type (lumen-less leads [LLLs], *n* = 20; stylet-driven leads [SDLs], *n* = 22). Procedural and pacing parameters were assessed through a 4-year follow-up. **Results:** In select patients with adequate acoustic windows, multi-stage TTE-assisted LBBAP remained feasible despite a 54.8% overweight/obesity rate. The scrubbed aseptic workflow showed significantly shorter procedural and fluoroscopy times (*p* < 0.01), though confounded by the learning curve. Beyond 6 months, pacing parameters remained stable and comparable between workflows through 4 years. Regarding lead types, procedural durations were similar. However, the SDL group exhibited significantly higher pacing thresholds at implant and 6 months (*p* < 0.05) and maintained a higher numerical trend through Year 4, though both lead types demonstrated stable chronic sensing and impedance profiles. No late dislodgements, device-related infections, or late complications occurred across available patient-years. **Conclusions:** TTE-assisted LBBAP is a clinically feasible approach that can supplement conventional implantation in selected patients with favorable acoustic windows. While achieving excellent 4-year functional durability, procedural improvements reflect the combined effect of workflow refinement and operator proficiency.

## 1. Introduction

Cardiac implantable electronic devices are widely used to treat a variety of cardiac arrhythmias worldwide, with approximately one million pacemakers implanted annually [1]. Conventional right ventricular (RV) pacing may result in ventricular desynchrony-related reduced cardiac output and cardiac dysfunction as pacemaker-induced cardiomyopathy. By contrast, left bundle branch area pacing (LBBAP) is an emerging physiologic pacing strategy in which a pacing lead is advanced through the RV septum into the left ventricular septum to capture the left bundle branch of the cardiac conduction system, thereby reducing the risk of pacemaker-induced cardiomyopathy and heart failure hospitalization [2,3,4,5,6].

Fluoroscopic guidance remains the standard imaging modality for LBBAP implantation; however, it inevitably exposes both patients and operators to ionizing radiation, often to a greater extent than conventional RV pacing [7,8,9,10]. Several non-fluoroscopic alternatives, including electromagnetic mapping systems, intracardiac echocardiography, and transthoracic echocardiography (TTE), have been explored [1,11,12,13,14,15]. TTE is a non-invasive, convenient, and reproducible imaging modality that may reduce radiation exposure and facilitate early detection of procedural complications compared with standard fluoroscopy [16,17]. Previous studies have demonstrated that TTE-assisted LBBAP enables accurate septal lead positioning, achieves high implantation success rates, and provides more favorable mid-term pacing outcomes [12,18,19].

However, the optimal clinical workflow for integrating echocardiographic monitoring into the implantation environment has not been fully established. Therefore, this retrospective study aimed to evaluate the feasibility and long-term parameter stability of TTE-assisted, low-fluoroscopy LBBAP in selected patients with favorable acoustic windows. Specifically, we evaluated an experience conducted by a single operator comparing two sequential workflows and two distinct lead types.

## 2. Material and Methods

### 2.1. Study Design

This study was conducted at Mackay Memorial Hospital (MMH), approved by the Institutional Review Board of MMH (IRB: 22MMHIS358e). Clinical data from medical charts of patients undergoing TTE-assisted LBBAP from November 2021 to June 2022 were analyzed. This study was carried out in accordance with the applicable local regulations and the ethical principles of the Declaration of Helsinki. As a retrospective study, the requirement for informed consent was waived.

### 2.2. Patients

This retrospective study included 45 consecutive patients aged ≥18 years who underwent TTE-assisted LBBAP using either a lumen-less pacing lead (LLL; SelectSecure 3830, Medtronic, Minneapolis, MN, USA) or a stylet-driven lead (SDL; Solia S60, Biotronik SE & Co. KG, Berlin, Germany). Clinical and device-related parameters were assessed at 6 months and annually thereafter for up to 4 years following pacemaker implantation. The selection of the pacing lead was based on the implanting physician’s or the patient’s preference, as well as the availability of leads and implantation tools. Patients were excluded from the study if they had incomplete medical records regarding fluoroscopic time, procedural time, paced electrocardiogram (ECG) characteristics, or pacing parameters.

### 2.3. Study Procedure

**Figure 1:**
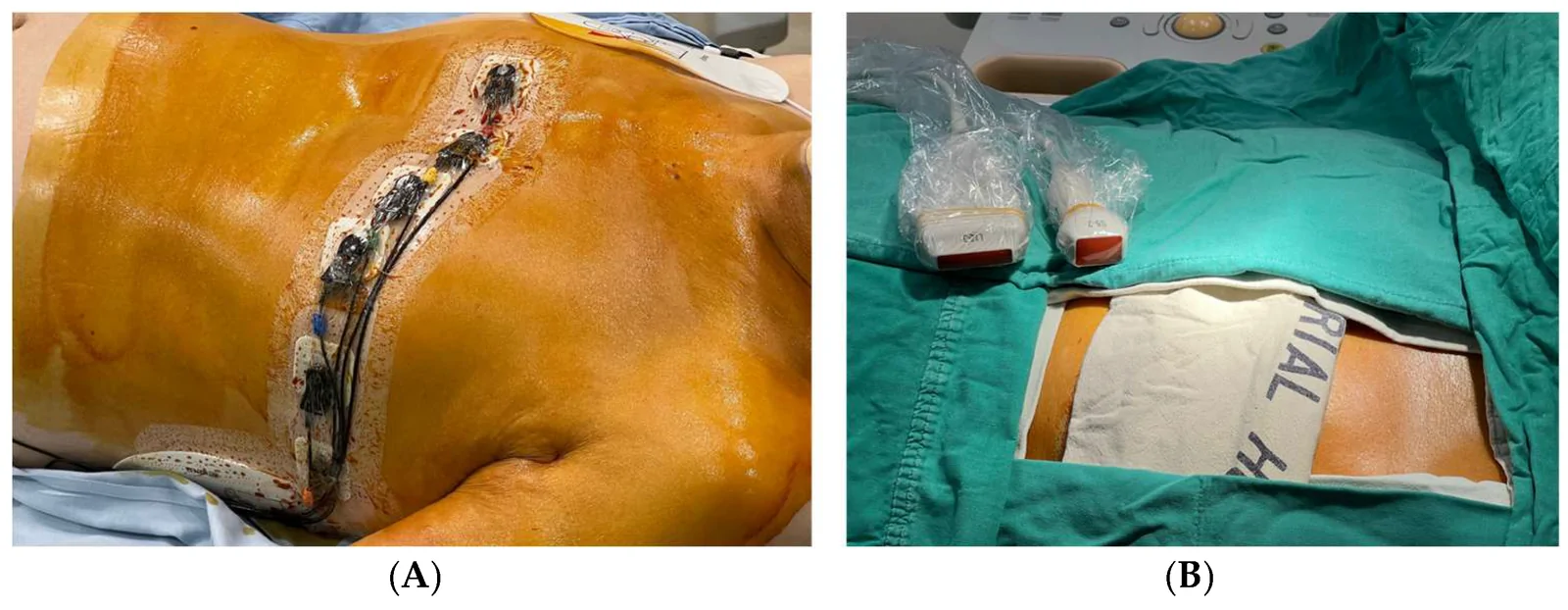
Patient preparation and modified sterile draping for scrubbed aseptic TTE-assisted LBBAP. (**A**) Precordial surface ECG electrodes and cables were covered with sterile transparent adhesive film after skin preparation, allowing continuous ECG monitoring within the procedural field. (**B**) Modified sterile draping of the left neck and chest wall provided an acoustic window for echography image acquisition. The ultrasound probe was enclosed in a sterile plastic sleeve, and sterile gel was applied directly to the prepared skin. ECG, electrocardiography; TTE, transthoracic echocardiography; LBBAP, left bundle branch area pacing.

All surgical procedures were performed by the same electrophysiologist (S-H C), with assistance from fellows and clinical staff for echocardiography. Antibiotic prophylaxis was administered immediately before the procedure. Patients were positioned in the supine position. The optimal echocardiographic acoustic windows for delineating the interventricular septum were first identified. Precordial leads of the 12-lead surface ECG were covered with sterile transparent film (Tegaderm, 3M, Maplewood, MN, USA). The left neck and chest wall were cleaned, and the surgical draping process was modified to allow acquisition of TTE images (Figure 1). The ultrasound machine was positioned on the side opposite to the operator. Under local anesthesia, an ultrasound-assisted left axillary vein approach was used to obtain two venous accesses, thereby avoiding X-ray exposure and contrast administration. Two different workflows were employed to acquire cardiac images for LBBAP. The non-scrubbed echocardiography-assisted approach was applied to the initial 24 patients. Subsequently, the scrubbed aseptic approach was used for the following 18 patients. In the non-scrubbed echocardiography-assisted approach, the fellow was not scrubbed, stood on the side opposite to the operator, and obtained images beneath the sterile drape. In the scrubbed aseptic technique, the fellow was scrubbed and stood on the same side as the operator. The ultrasound probe was enclosed in a sterile plastic sleeve, and sterile gel was applied directly to the skin to acquire cardiac images (Figure 1). To ensure maximum occupational radiation safety, a strict alternating, multi-stage imaging workflow was implemented. The echocardiography operator performed TTE monitoring only during periods when fluoroscopy was inactive. Whenever fluoroscopy was engaged by the primary implanting team, the echocardiography operator completely retracted their hands from the patient’s thorax and stepped away from the radiation field into a shielded safe zone. The operators wore standard personal protective equipment (lead apron and thyroid shield) with body dosimeter monitoring. Consequently, direct or scatter radiation exposure to the echocardiography operator’s hands was systematically avoided throughout the procedure.

For the LLL implantation, a 23 cm peel-away 7F sheath (Medtronic Inc., Minneapolis, MN, USA) was inserted into the left axillary vein, and a fixed double-curve delivery sheath (C315 His, Medtronic Inc., Minneapolis, MN, USA) was employed. For SDL implantation, the electrically active helix was pre-extended. The green stylet-guide tool was connected to the pin, rotated 8–10 times clockwise, and maintained on the lead pin during lead insertion. This process increased tension on the inner coil and helped prevent helix retraction during lead rotation. A pre-shaped sheath (Selectra 3D, Biotronik, SE & Co., KG, Germany) was used to deliver the lead [20,21,22,23].

**Figure 2:**
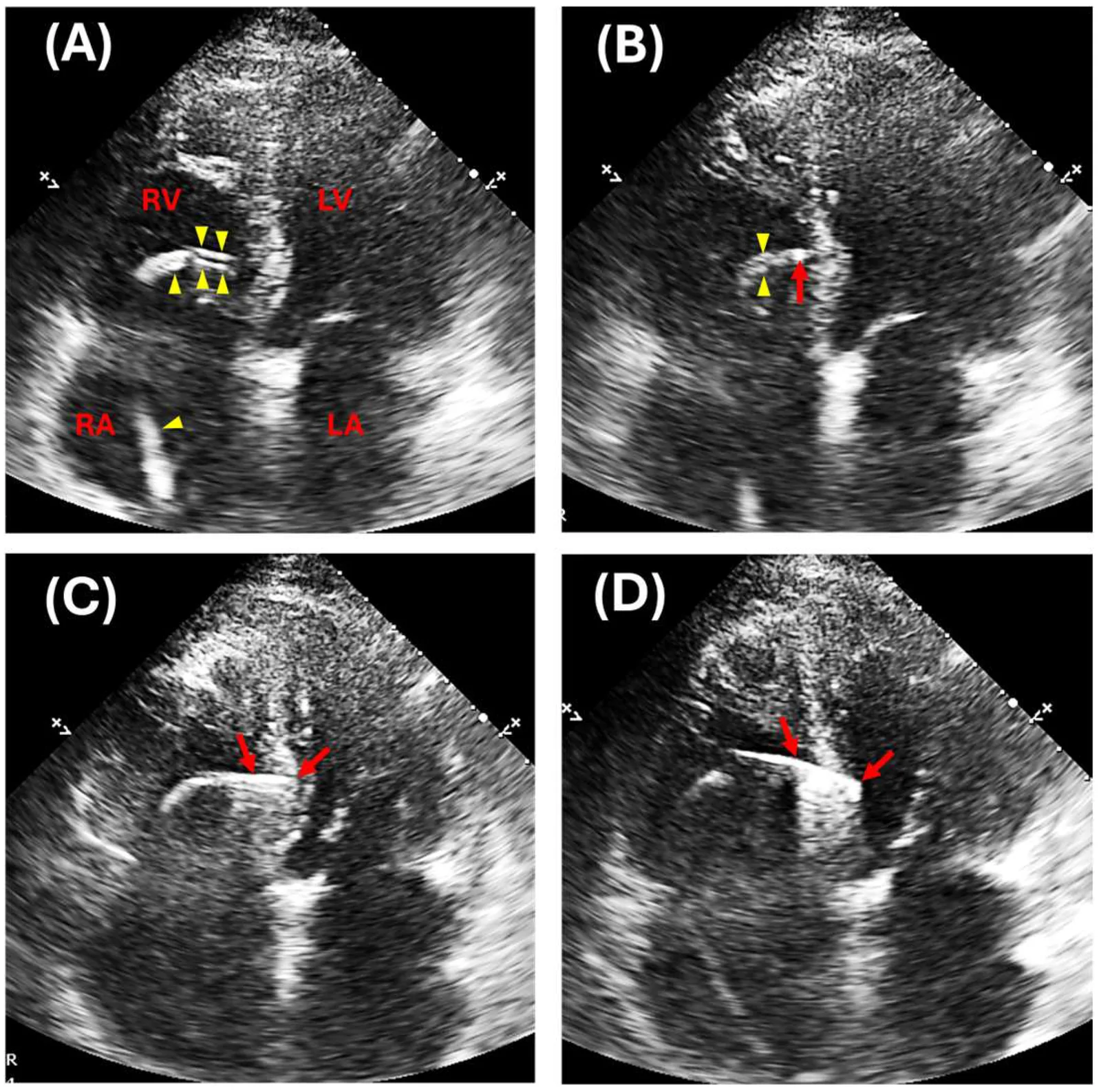
Sequential echocardiographic monitoring of LBBAP lead deployment in the apical 4-chamber view. (**A**) General visualization of the LBBAP delivery sheath (yellow arrowheads) aligned against the right ventricular endocardial septum. The delivery system exhibits a characteristic double-rail appearance characterized by parallel hyperechoic margins and a central anechoic lumen. (**B**) Visualization of a lead-related echogenic structure (red arrow) advancing through the lumen of delivery sheath (yellow arrowheads). (**C**) Visualization of the lead-related echogenic structure (red arrows) penetrating into the interventricular septum, with the apparent trajectory of the dense hyperechoic line tracked traversing the mid-myocardial tissue. (**D**) Final multi-stage visualization showing the terminal portion of the lead-related echogenic structure= (red arrows) near the left ventricular endocardial surface. LBBAP, left bundle branch area pacing; RV, right ventricle; LV, left ventricle; RA, right atrium; LA, left atrium.

**Figure 3:**
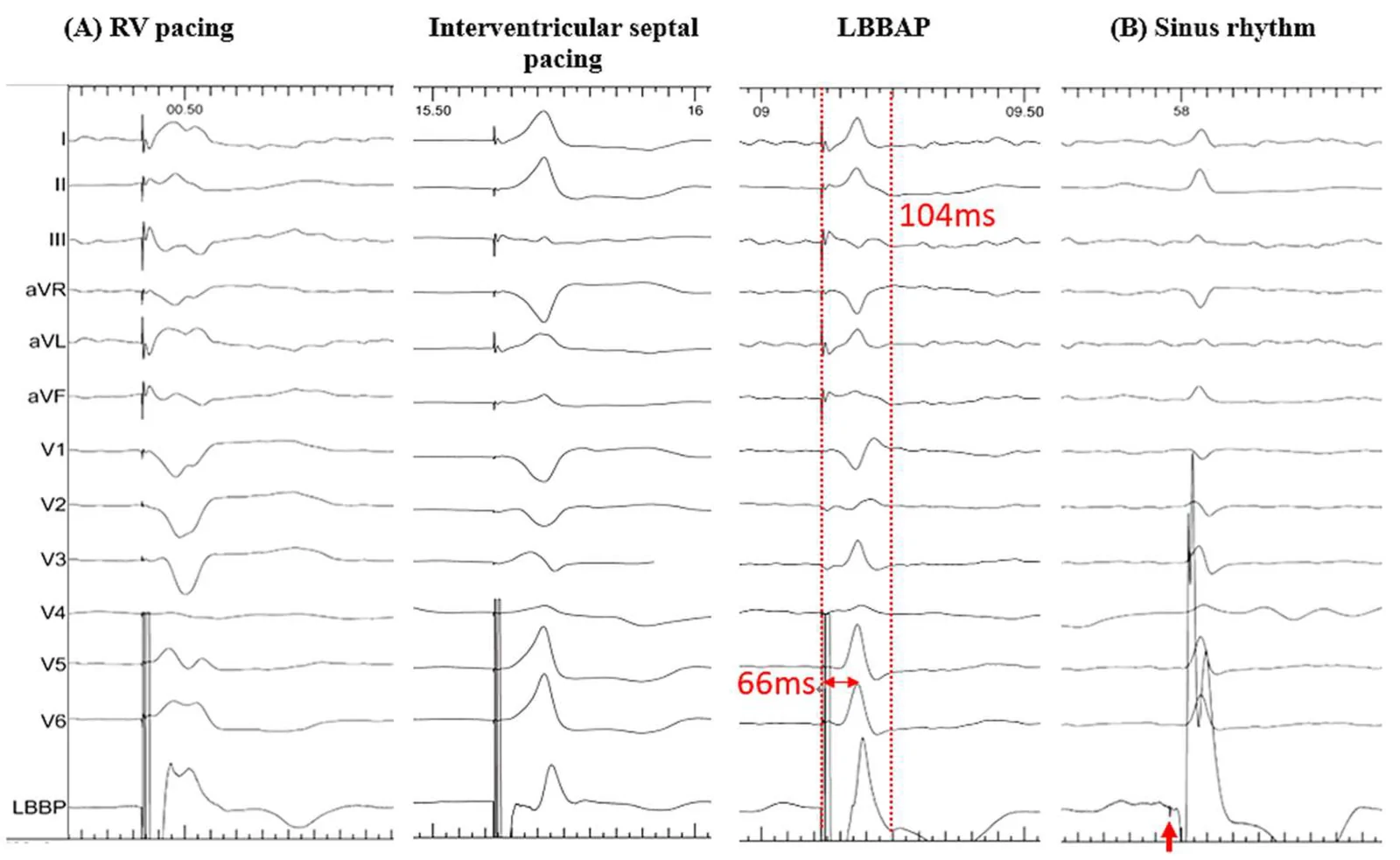
Twelve-lead surface electrocardiograms and intracardiac electrograms recorded from the LBBAP lead. (**A**) From left to right, serial recordings demonstrate changes in paced QRS morphology during progression from RV septal pacing through interventricular septal pacing to LBBAP. As the lead was advanced from the RV septal endocardium toward the deep septal region adjacent to the LV subendocardium, the paced QRS morphology in lead V1 changed from a “W” pattern to a “Qr” pattern. During LBBAP, the V6 R-wave peak time (red double-headed arrow) was 66 ms, and the paced QRS duration (red dashed lines) was 104 ms. (**B**) The intracardiac electrogram recorded during sinus rhythm demonstrated a left bundle branch potential (arrow). RV, right ventricle; LBBP, left bundle branch pacing; LBBAP, left bundle branch area pacing.

The delivery sheath was advanced into the right ventricle via a J-tip guidewire and visualized using TTE or brief fluoroscopy. The pacing lead was then introduced through the sheath and positioned at the basal or mid-RV septum. In this position, pace mapping with unipolar pacing at the lead tip typically produced a wide “W”-shaped QRS morphology in lead V1, along with a positive-to-isoelectric lead II and an isoelectric-to-negative lead III. The lead tip was advanced into the septum by clockwise rotation of the outer lead body toward the left ventricular (LV) septal endocardium under TTE monitor in the apical four-chamber or parasternal view (Figure 2). As the lead reached the LV endocardium, the paced “W”-shaped QRS morphology in lead V1 changed to a narrow “Qr or rSR” shaped QRS morphology (Figure 3). Successful LBBAP was defined by the appearance of a right bundle branch block (RBBB) pattern in lead V1 and a shortened V6 R-wave peak time (V6RWPT), or by presence of an LBB potential on the unipolar lead-tip electrogram [4,24,25]. A standard atrial bipolar active-fixation pacing lead was positioned in the right atrial appendage under fluoroscopic guidance. The pacemaker settings were optimized, and the leads were secured with suture ligatures. The final lead location and lead slack were confirmed using post-procedure fluoroscopy.

Clinical data including patients’ demographic characteristics, medical histories, and pacing indications, were collected from medical charts. Pacing characteristics such as sensing amplitude, pacing thresholds, and lead impedances were recorded immediately after implantation, at 6 months and annually through 4 years post-implantation. Total procedural time was defined as the duration from axillary vein puncture to completion of subcutaneous suture. For LBBAP, the procedural and fluoroscopic time were measured from advancement of delivery sheath to the successful achievement of LBBAP. Paced QRS duration was measured from the onset to the end of QRS complex. The V6RWPT was measured from pacing stimulus to the peak of the R wave in leads V6 during unipolar LBBAP. Peri- and post-procedural device-related complications, including pericardial effusion, pneumothorax, lead dislodgement, lead perforation, infection, and pocket hematoma, were documented.

### 2.4. Statistical Analysis

Descriptive statistics were used to present the categorical data as numbers and percentages, and continuous data as numbers, medians, ranges, and standard deviations (SD). In addition to describing the overall population, outcomes were also compared between patients with different types of techniques and leads. Continuous variables were compared using Student’s *t*-test or the Mann–Whitney U test, and categorical variables were compared using Chi-square test or Fisher’s exact test. CUSUM analysis for LBBAP procedure and fluoroscopy time was performed to evaluate the surgical learning curve and assess the chronological attainment of procedural proficiency [26]. A *p*-value of less than 0.05 was considered statistically significant. All analyses were performed using Statistical Package for the Social Sciences (SPSS, version 25, Chicago, IL, USA).

## 3. Results

### 3.1. Baseline Characteristics

A total of 45 patients were initially enrolled for TTE-assisted LBBAP. However, the procedure could not be completed under TTE monitor in three patients. In one patient with hypertrophic cardiomyopathy, successful fixation of the pacing lead within the left ventricular septum could not be achieved due to structural constraints, and the procedure was therefore converted to conventional right ventricular pacing. The remaining two patients were converted to fluoroscopic guidance because of inadequate cardiac image quality precluded safe TTE visualization of the lead trajectory. Specifically, the poor acoustic windows in these two cases were attributed to hyperinflated lungs from chronic obstructive pulmonary disease (COPD) in one patient, and retrosternal tissue distortion from previous coronary artery bypass grafting (CABG) in the other. These three converted cases were explicitly accounted for in the total cohort, yielding an overall TTE-assisted procedural success rate of 93.3% (42/45).

The mean body mass index (BMI) of the cohort was 25.3 ± 4.2 kg/m^2^. Overweight individuals (BMI ≥ 25 kg/m^2^) constituted 54.8% (*n* = 23) of the study population, and 31.0% (*n* = 13) were classified as obese (BMI ≥ 27 kg/m^2^). Additional baseline clinical characteristics are summarized in Table 1. 

There were no significant differences in demographic or baseline clinical characteristics between the two techniques, indicating comparable baseline conditions. The prevalence of comorbidities, including hypertension, diabetes mellitus, chronic kidney disease, coronary artery disease, valvular heart disease, atrial fibrillation, heart failure, and cerebrovascular disease, was similar between groups, with the exception of a lower prevalence of persistent atrial fibrillation in the scrubbed aseptic group.

### 3.2. Procedural Characteristics of Patients Undergoing Different Workflows of Echocardiographic Assistance

The use of the scrubbed aseptic approach during implantation was associated with significant reductions in LBBAP procedural time, total procedural time, total fluoroscopy time, and LBBAP fluoroscopy time compared with the non-scrubbed echocardiography-assisted approach (Table 2). Total fluoroscopy time was significantly shorter in the scrubbed aseptic group (88.3 ± 50.5 s) than in the non-scrubbed group (141.8 ± 94.1 s; *p* = 0.035). Similarly, LBBAP fluoroscopy time was markedly reduced with the scrubbed aseptic approach (32.2 ± 24.9 s) compared with the non-scrubbed approach (97.2 ± 75.6 s; *p* = 0.001). Total procedural time was also significantly shorter in the scrubbed aseptic group (74.4 ± 17.8 min) than in the non-scrubbed group (90.8 ± 29.7 min; *p* = 0.046). In addition, LBBAP procedural time was significantly reduced with the scrubbed aseptic group (22.4 ± 13.6 min vs. 39.6 ± 21.7 min; *p* = 0.005).

**Figure 4:**
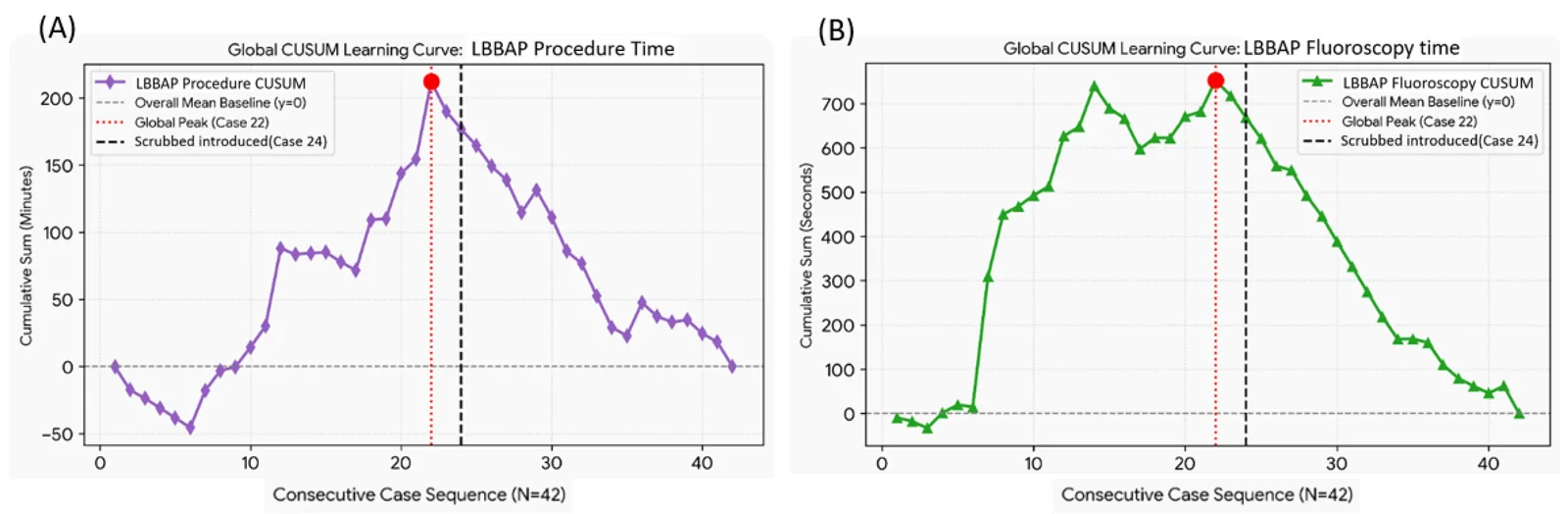
Cumulative Sum (CUSUM) learning-curve analysis for successfully completed TTE-assisted LBBAP cases. (**A**) CUSUM curve for LBBAP procedural time. (**B**) CUSUM curve for LBBAP fluoroscopy time. For both efficiency metrics, the global peak (indicated by the red circle) is syn-chronously achieved at Case 22, denoting the precise chronological turning point where the oper-ator attained procedural proficiency. Notably, this learning-curve inflection point preceded the transition from the non-scrubbed echocardiography-assisted workflow to the scrubbed aseptic workflow (indicated by the vertical dashed line at Case 24) by two cases. This chronological se-quence statistically demonstrates that while a background training effect was present, the subse-quent cliff-style reductions in both procedural and fluoroscopy durations were heavily amplified by the structural advantages introduced by scrubbed aseptic workflow. LBBAP, left bundle branch area pacing.

The CUSUM analysis (*N* = 42, Figure 4) was performed to clarify the efficiency gains with the operator’s learning curve. The LBBAP procedural and fluoroscopy times reached a synchronized learning-curve inflection point at case 22, which preceded the transition to scrubbed aseptic workflow at case 24. Notably, the primary efficiency metrics exhibited an abrupt, discontinuous decrease following the workflow transition. When the workflow changed from the non-scrubbed to the scrubbed echocardiography-assisted approach, the LBBAP fluoroscopy time demonstrated a corresponding step-like contraction. The mean LBBAP fluoroscopy time decreased steeply from 97.2 ± 75.6 s to 32.2 ± 24.9 s (*p* = 0.001), reflecting a substantial 67% reduction. The mean LBBAP procedural time dropped sharply from 39.6 ± 21.7 min to 22.4 ± 13.6 min (*p* = 0.005). This sudden, simultaneous reduction suggests an additive operational leap rather than a purely incremental training effect.

### 3.3. Pacing Characteristics and Long-Term Follow-Up After Implantation Using Different Workflow Strategies

**Figure 5:**
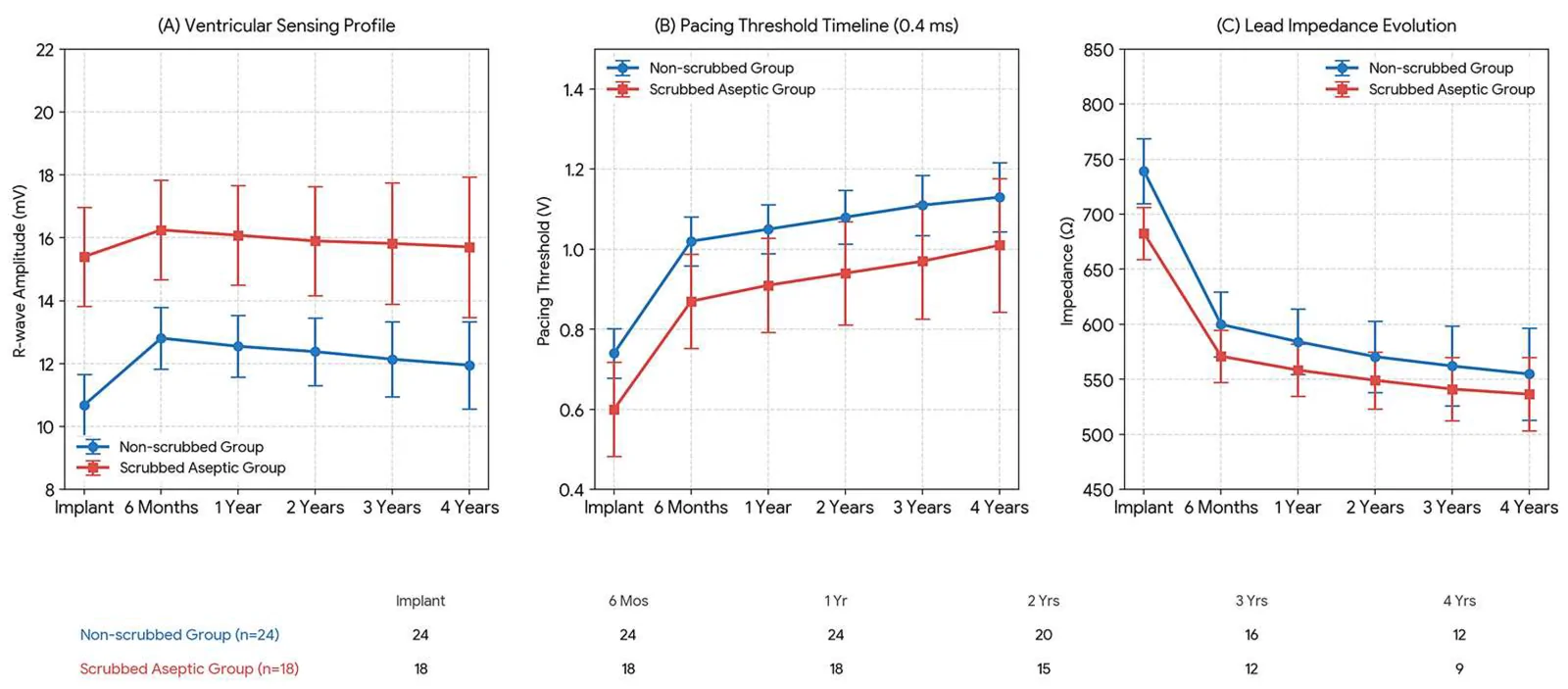
Chronological pacing parameter profiles and available long-term follow-up patient counts up to 4 years. Longitudinal comparisons between the non-scrubbed echocardiography-assisted group (blue circles, baseline successful *n* = 24) and the scrubbed aseptic group (red squares, baseline successful *n* = 18) demonstrate highly reliable pacemaker parameter maintenance across a 4-year follow-up spectrum. (**A**) Ventricular sensing (R-wave amplitude), (**B**) Ventricular pacing thresholds at 0.4 ms, and (**C**) Ventricular lead impedance evolution all show physiologic stabilization and hardware durability without late failure peaks. Data points indicate mean ± standard error of the mean. The bottom panel delineates the exact number of patients with available clinical measurements at each distinct year.

To assess the impact of different TTE-assisted approaches, pacing parameters were analyzed longitudinally at implantation, 6 months, and annually up to 4 years (Table 3 and Figure 5). At implantation, the scrubbed aseptic group exhibited significantly higher sensed R-wave amplitudes (15.4 ± 6.7 mV vs. 10.5 ± 4.8 mV; *p* = 0.009) compared with the non-scrubbed group. Both pacing thresholds and lead impedance were numerically lower in the scrubbed aseptic group (0.6 ± 0.2 V, 682.7 ± 100.2 Ω) compared with the non-scrubbed group (0.8 ± 0.2 V, 739.3 ± 145.0 Ω), although this difference did not reach statistical significance (*p* > 0.05).

Longitudinal follow-up data were collected up to 4 years. Owing to the retrospective study design and varying individual follow-up durations, available 4-year data were limited to 9 patients in the scrubbed aseptic group and 12 patients in the non-scrubbed echocardiography-assisted group. From 6 months onward, sensed R-wave amplitudes were consistently higher in the scrubbed aseptic group than in the non-scrubbed group (Figure 5). Pacing thresholds exhibited a physiologic chronic upward drift, with no significant differences between the cohorts at year 4 (scrubbed aseptic group: 1.0 ± 0.4 V; non-scrubbed group: 1.1 ± 0.3 V). Concurrently, lead impedance profiles evolved identically across both groups, characterized by an expected post-implantation drop to approximately 570–600 Ω at 6 months, followed by chronic maintenance between 530 and 560 Ω through the 4-year horizon.

### 3.4. Procedural and Pacing Characteristics in Patients Using Different Types of Leads

Baseline characteristics were generally comparable between patients receiving lumen-less leads (LLLs) and stylet-driven leads (SDLs). No significant differences were observed in demographic variables, pacing indications, or most comorbidities, with the exception of a higher prevalence of cerebrovascular accident in the SDL group (*p* = 0.045). The distribution of workflow techniques was also similar between the LLL and SDL groups (Table 4). Procedural characteristics, including procedure time, number of rotation attempts, and fluoroscopy duration, were comparable between the LLL and SDL groups (Supplementary Table S1).

At implantation, the SDL group was associated with a significantly higher ventricular pacing threshold (0.9 ± 0.3 V vs. 0.6 ± 0.5 V, *p* = 0.018) and lower lead impedance (*p* = 0.047) compared with the LLL group. Other pacing parameters, including ventricular sensing amplitudes and paced QRS durations, did not differ significantly between groups (Table 5).

From 6 months onward, pacing thresholds were higher in the SDL group than in the LLL group; however, this difference was statistically significant only at 6 months (1.0 ± 0.2 V vs. 0.8 ± 0.3 V, *p* = 0.048) and dissolved into a non-significant numerical trend by Year 4 (1.3 ± 0.6 V vs. 1.0 ± 0.4 V, *p* = 0.143) due to the reduced sample size. No significant between-group differences were observed in ventricular sensing or lead impedance over 4 years. Both groups demonstrated a parallel physiologic decline in impedance, consistent with stable chronic lead positioning. Overall, although chronic pacing thresholds rose gradually and remained higher with the SDL approach, both lead strategies demonstrated functionally stable and clinically acceptable durability over 4 years.

### 3.5. Complications

Throughout the 4-year follow-up, there were no cases of septal perforation into the left ventricular cavity, late lead dislodgement, device-related infection, macrodisplacement, pericardial effusion, pneumothorax, pacing-induced cardiomyopathy or catastrophic threshold spikes. These findings reinforced the long-term electrical safety and mechanical integrity of both technical approaches and both lead types.

## 4. Discussion

To our knowledge, this is the first study to evaluate a TTE-assisted, low-fluoroscopy LBBAP experience in selected patients with favorable acoustic windows, comparing two sequential probe-handling workflows (a non-scrubbed echocardiography-assisted approach vs. a scrubbed aseptic approach) and two lead types (lumen-less vs. stylet-driven leads). The overall technical success rate for TTE-assisted LBBAP was 93.3% (42/45 cases). Ultimately, we demonstrated the feasibility and 4-year functional durability of both workflows and both lead types when utilizing multi-stage TTE as a supplement to conventional fluoroscopy.

The key findings of this study are as follows: (1) the scrubbed aseptic approach was associated with significantly shorter total procedure and fluoroscopy times compared with the non-scrubbed echocardiography-assisted approach; (2) the scrubbed aseptic approach yielded higher ventricular sensing amplitudes and lower pacing thresholds at the time of implantation; the sensed R-wave amplitudes were consistently higher in the scrubbed aseptic approach through a 4-year follow-up; and (3) long-term procedural and electrical outcomes were comparable between LLLs and SDLs when LBBAP was performed with supplementary TTE monitoring.

Although our cohort demonstrated a high technical success rate, TTE-assisted LBBAP relies heavily on the quality of the acoustic window; thus, our findings are inherently biased toward patients with favorable cardiac anatomy. In this study, patients with severe COPD or a history of CABG experienced profound acoustic impairment. This necessitated immediate conversion to conventional fluoroscopy-guided pacing. However, it is noteworthy that this anatomical limitation was not strictly dictated by body habitus. The mean BMI of the successfully implanted cohort was 25.3 ± 4.2 kg/m^2^, with overweight individuals (BMI ≥ 25 kg/m^2^) constituting 54.8% (*n* = 23) and obese individuals (BMI ≥ 27 kg/m^2^) accounting for 31.0% (*n* = 13). Despite this high prevalence of overweight and obesity, TTE remained highly feasible and provided allowable anatomical visualization across this broad anthropometric spectrum in the supine procedural position. Therefore, while our findings demonstrate that elevated BMI alone is not an absolute barrier to this approach, the prerequisite for an acceptable acoustic window remains a critical selection bias that limits the universal generalizability of this technique.

The clinical utility of the TTE-assisted approach lies primarily in its role as a supplementary tool for gross anatomical confirmation and intraoperative safety. Although standard LBBAP relies heavily on fluoroscopic and electrophysiological endpoints, these parameters do not provide direct visualization of myocardial architecture or the spatial relationship between the lead-sheath assembly and the endocardial surface. In our protocol, TTE offered supplementary anatomical context prior to active lead fixation. By confirming a gross perpendicular alignment of the delivery sheath against the right ventricular endocardial septum (Figure 2A) and allowing a relative visual estimation of septal thickness, the implanter gained essential structural confidence before initiating deep septal rotation. Crucially, the incidence of permanent left ventricular septal perforation and acute pericardial effusion was 0% among the 45 consecutive patients attempted.

Based on these favorable outcomes, the scrubbed aseptic approach officially became the routine clinical standard at our center since June 2022. To resolve the technical imaging constraints identified in our early experience, we refined the procedural protocol by removing the chest electrocardiogram leads V2, V3, and V4. This simple modification significantly unobstructed the acoustic window, rendering the parasternal views substantially easier to manipulate and further improving our subsequent institutional success rate. Furthermore, while instances of transient intraoperative septal perforation did occur during our extended clinical experience beyond June 2022, this optimized TTE-assisted workflow served as an invaluable decisional tool. It provided immediate visual evidence of the perforation, thereby guiding prompt, real-time lead repositioning without any permanent clinical sequelae or pericardial effusion. This clinical experience strongly suggests that while TTE operates alongside conventional electrical mapping, it provides useful, non-invasive anatomical feedback that effectively helps manage major mechanical complications during deep septal deployment.

Consistent with prior studies, our results demonstrated a significant reduction in fluoroscopy time with TTE-assisted LBBAP approach [18,19]. Notably, the scrubbed aseptic approach further reduced fluoroscopy exposure to less than 1 min. Compared with conventional fluoroscopy-guided approaches, this substantial reduction in radiation exposure may benefit both patients and operators by lowering the risk of radiation-associated malignancy and other adverse effects. On the other hand, prolonged procedure duration may increase the risk of infection and patient discomfort. While previous studies reported comparable procedure times between TTE-assisted and fluoroscopy-guided approaches [18], we observed a further reduction in total procedure time with the scrubbed aseptic approach compared with the non-scrubbed echocardiography-assisted approach, which may potentially reduce infection risk.

However, these marked temporal differences between the two sequential workflows must be interpreted with caution, as they are inherently subject to a substantial learning transfer effect. The technical experience gained during the initial 24 cases of the non-scrubbed echocardiography-assisted approach undoubtedly established a foundational skill set that directly benefited the subsequent 18 cases performed via the scrubbed aseptic approach. However, while incremental learning typically yields gradual time reductions, our data revealed an abrupt contraction in procedural metrics immediately following the workflow transition. Because this disproportionate and concurrent reduction cannot be mathematically explained solely by progressive learning, it suggests that the scrubbed aseptic approach introduces inherent structural and ergonomic advantages, such as simplifying intraoperative steps, optimizing instrument handling, and eliminating non-sterile team communication loops, that effectively amplified the background training effect. Nevertheless, due to the sequential study design, this temporal confounding represents a primary chronological limitation.

Our study also demonstrated that both SDLs and LLLs are suitable for TTE-assisted LBBAP. Previous meta-analyses have shown that SDLs are associated with higher pacing thresholds at implantation, with no significant differences at 1- and 6-month follow-up. This early difference has been attributed to the larger helix and lead body diameter of SDLs, which may cause greater local myocardial trauma during septal penetration [27,28,29,30]. In our TTE-assisted LBBAP cohort, the SDL group exhibited significantly higher pacing thresholds than the LLL group at both implantation and 6-month follow-up and maintained a higher numerical trend through Year 4. These findings suggest that lead-related myocardial trauma or localized micro-fibrosis associated with SDLs may persist for a longer duration under certain placement conditions, although both strategies ultimately yielded functionally stable and clinically manageable electrical performance throughout the 4-year follow-up.

### Study Limitations

This study has several limitations. First, it was a single-center, single-operator, retrospective study with a relatively small sample size, which inherently limit the generalizability of the findings and renders the study likely underpowered to detect clinically meaningful differences in pacing parameters or complications. Second, intraoperative TTE monitoring was performed by cardiology fellows in training, which may have introduced subtle variations in image quality and procedural efficiency. Nevertheless, the fluoroscopy times remained consistently low across the cohort. Third, given the retrospective nature of evaluating our early clinical experience, definitive left bundle branch capture was not electrophysiologically verified in all patients. Thus, the potential inclusion of deep left ventricular septal pacing cannot be entirely excluded. Consequently, the term LBBAP is used throughout this study to reflect this clinical population. Fourth, this protocol evaluates a TTE-assisted, low-fluoroscopy approach rather than a completely fluoroscopy-free technique, as fluoroscopy remained mandatory for right atrial lead placement and final position verification. Fifth, granular imaging endpoints, such as the exact duration of image loss, reacquisition time, and interobserver agreement, were not quantitatively recorded. Consequently, the TTE-assisted framework described herein represents an intermittent, cross-sectional visualization tool rather than an uninterrupted, true real-time tracking modality. Finally, potential predictors of severe acoustic window impairment, such as advanced COPD or atypical thoracic anatomy, were not systematically quantified in this cohort. The clinical applicability of this technique remains restricted to selected patients with favorable baseline acoustic windows. Future prospective, multicenter randomized trials utilizing standardized imaging protocols and conventional fluoroscopy control groups are warranted to establish precise selection criteria and validate the noninferiority of this low-fluoroscopy workflow.

## 5. Conclusions

TTE-assisted, low-fluoroscopy LBBAP is a clinically feasible approach that can supplement conventional implantation, particularly in select patients with optimal acoustic windows. While our experience shows promising procedural efficiency in a scrubbed workflow, these findings reflect a combined effect of technical optimization and learning-curve progression, rather than a standalone validation of TTE as a primary navigation modality.

## Figures and Tables

**Table 1:** Demographics and baseline characteristics in different echocardiography-assisted approaches.

Characteristics	Total *N* = 42	Non-Scrubbed *N* = 24	Scrubbed *N* = 18	*p*-Value ^†^
Demographics				
Mean age (years)	77.1 ± 10.1	79.6 ± 6.7	73.7 ± 12.8	0.246
Male sex, *n* (%)	18 (42.9)	11 (45.8)	7 (38.9)	0.653
Weight (kg)	62.7 ± 13.7	61.2 ± 11.5	64.5 ± 16.4	0.456
Height (cm)	157.0 ± 7.8	156.6 ± 8.1	157.5 ± 7.6	0.725
Body mass index (kg/m^2^)	25.3 ± 4.2	24.9 ± 3.2	25.8 ± 5.1	0.516
Pacing indication				
AV block	21 (50.0)	9 (37.5)	12 (66.7)	0.064
SSS	13 (31.0)	9 (37.5)	4 (22.2)	0.301
AF with SVR	8 (19.0)	6 (25.0)	2 (11.1)	0.268
Method of echo-guide, *n* (%)				
Lumen-less lead	20 (47.6)	9 (37.5)	11 (61.1)	0.129
Stylet-driven lead	22 (52.4)	15 (62.5)	7 (38.9)	0.129
Medical history, *n* (%)				
Hypertension	37 (88.1)	21 (87.5)	16 (88.9)	0.891
Diabetes mellitus	17 (40.5)	7 (29.2)	10 (55.6)	0.085
Chronic kidney disease	19 (45.2)	10 (41.7)	9 (50.0)	0.591
Coronary artery disease	14 (33.3)	7 (29.2)	7 (38.9)	0.508
Valvular heart disease	10 (23.8)	7 (29.2)	3 (16.7)	0.347
Paroxysmal atrial fibrillation	13 (31.0)	6 (25.0)	7 (38.9)	0.335
Persistent atrial fibrillation	9 (21.4)	8 (33.3)	1 (5.6)	0.030
HFrEF	4 (9.5)	3 (12.5)	1 (5.6)	0.448
Cerebrovascular accident	10 (23.8)	8 (33.3)	2 (11.1)	0.099

Data are presented as means ± SD or number(percentage). AV block, atrioventricular block; SSS, sick sinus syndrome; AF with SVR, atrial fibrillation with slow ventricular response; HFrEF, heart failure with reduced ejection fraction. ^†^ Comparison between non-scrubbed and scrubbed echocardiography-assisted approaches.

**Table 2:** Procedural characteristics in different echocardiography-assisted approaches.

Characteristics	Total *N* = 42	Non-Scrubbed *N* = 24	Scrubbed *N* = 18	*p*-Value ^†^
**Procedural characteristics**				
LBBAP procedure time (min)	32.3 ± 20.3	39.6 ± 21.7	22.4 ± 13.6	0.005
LBBAP attempts (times)	2.3 ± 1.1	2.5 ± 1.1	2.1 ± 1.0	0.158
LBBAP fluoroscopic time (s)	69.4 ± 67.5	97.2 ± 75.6	32.2 ± 24.9	0.001
Total procedure time (min)	83.8 ± 26.3	90.8 ± 29.7	74.4 ± 17.8	0.046
Total fluoroscopic time (s)	118.8 ± 82.1	141.8 ± 94.1	88.3 ± 50.5	0.035

Data are presented as means ± SD; LBBAP, left bundle branch area pacing. ^†^ Comparison between non-scrubbed and scrubbed echocardiography-assisted approaches.

**Table 3:** Pacing characteristics at implantation in different echocardiography-assisted approaches.

Characteristics	Total *N* = 42	Non-Scrubbed *N* = 24	Scrubbed *N* = 18	*p*-Value ^†^
**Pacing characteristics at implant**				
V6RWPT (ms)	76.1 ± 8.3	75.9 ± 8.0	76.4 ± 8.9	0.845
Paced QRS duration (ms)	116.1 ± 11.4	116.8 ± 12.6	115.2 ± 9.8	0.661
Ventricular—sense (mV)	12.6 ± 6.1	10.5 ± 4.8	15.4 ± 6.7	0.009
Ventricular—pacing threshold at 0.4 ms (V)	0.7 ± 0.2	0.8 ± 0.2	0.6 ± 0.2	0.097
Ventricular—lead impedance (Ω)	715.0 ± 129.5	739.3 ± 145.0	682.7 ± 100.2	0.163
Atrial—sense (mV)	3.3 ± 1.5	3.1 ± 1.6	3.5 ± 1.3	0.449
Atrial—pacing threshold at 0.4 ms (V)	0.6 ± 0.5	0.7 ± 0.5	0.5 ± 0.5	0.339
Atrial—lead impedance (Ω)	525.2 ± 74.8	521.4 ± 68.0	530.1 ± 84.3	0.716

Data are presented as means ± SD; V6RWPT, V6 R-wave peak time. ^†^ Comparison between non-scrubbed and scrubbed echocardiography-assisted approaches.

**Table 4:** Demographics and baseline characteristics in different types of leads.

Characteristics	Total *N* = 42	Lumen-Less Lead *N* = 20	Stylet-Driven Lead *N* = 22	*p*-Value ^†^
**Demographics**				
Mean age (years)	77.1 ± 10.1	76.0 ± 12.5	78.1 ± 7.4	0.499
Male sex, *n* (%)	18 (42.9)	9 (45)	9 (41)	0.789
Weight (kg)	62.7 ± 13.7	64.3 ± 16.7	61.3 ± 10.8	0.494
Height (cm)	157.0 ± 7.8	157.6 ± 8.	156.6 ± 7.4	0.697
Body mass index (kg/m^2^)	25.3 ± 4.2	25.6 ± 5.2	25.0 ± 3.0	0.642
**Surgical Procedure Techniques**				
Non-aseptic	24 (57.1)	9 (45.0)	15 (68.2)	0.136
Aseptic	18 (42.9)	11 (55.0)	7 (31.8)	0.138
**Pacing indication, *n* (%)**				
AV block	21 (50)	11 (55)	10 (45)	0.537
SSS	13 (31)	5 (25)	9 (41)	0.286
AF with SVR	8 (19)	4 (20)	3 (14)	0.591
**Medical history, *n* (%)**				
Hypertension	37 (88)	18 (90)	19 (86)	0.716
Diabetes mellitus	17 (40)	8 (40)	9 (41)	0.952
Chronic kidney disease	19 (45)	9 (45)	10 (46)	0.976
Coronary artery disease	14 (33)	7 (35)	7 (32)	0.827
Valvular heart disease	10 (24)	6 (30)	4 (18)	0.369
Paroxysmal atrial fibrillation	13 (31)	8 (40)	5 (23)	0.227
Persistent atrial fibrillation	9 (21)	5 (25)	4 (18)	0.591
HFrEF	4 (10)	1 (5)	3 (14)	0.341
Cerebrovascular accident	10 (24)	2 (10)	8 (36)	0.045

Abbreviation: AV block, atrioventricular block; SSS, sick sinus syndrome; AF with SVR, atrial fibrillation with slow ventricular response; HFrEF, heart failure with reduced ejection fraction. ^†^ Comparison between lumen-less pacing lead and standard stylet-driven lead.

**Table 5:** Pacing characteristics at implantation in different types of leads.

Characteristics	Total *N* = 42	Lumen-Less Lead *N* = 20	Stylet-Driven Lead *N* = 22	*p*-Value ^†^
**Pacing characteristics at implant**				
V6RWPT (ms)	76.1 ± 8.3	76.4 ± 8.9	75.9 ± 8.0	0.845
Paced QRS (ms)	116.1 ± 11.4	115.2 ± 9.8	116.8 ± 12.6	0.661
Ventricular—sense (mV)	12.6 ± 6.13	14.0 ± 7.4	11.5 ± 4.5	0.191
Ventricular—pacing threshold V at 0.4 ms (V)	0.8 ± 0.4	0.6 ± 0.5	0.9 ± 0.3	0.018
Ventricular—lead impedance (Ω)	715.0 ± 129.5	756.3 ± 124.3	677.5 ± 125.1	0.047
Atrial—sense (mV)	3.3 ± 1.5	3.3 ± 1.2	3.3 ± 1.7	0.872
Atrial—pacing threshold at 0.4 ms (V)	0.6 ± 0.5	0.4 ± 0.5	0.7 ± 0.5	0.098
Atrial—lead impedance (Ω)	525.2 ± 74.8	512.3 ± 79.0	536.3 ± 70.9	0.312

Data are presented as means ± SD; V6RWPT, V6 R-wave peak time. † Comparison between lumen-less pacing lead and standard stylet-driven lead.

## Data Availability

The original contributions presented in this study are included in the article. Further inquiries can be directed to the corresponding author.

## Supplementary Materials

The following supporting information can be downloaded at https://www.mdpi.com/article/10.3390/diagnostics16193172/s1. Table S1. Procedural characteristics in different types of leads.
