# Supplementary material for: Transthoracic Echocardiography-Assisted, Low-Fluoroscopy Left Bundle Branch Area Pacing: A Comparison of Implantation Approaches and Lead Types
Source: Diagnostics (Basel). 2026 Sep 29;16(19):3172. doi: 10.3390/diagnostics16193172 (PMC13664582; doi:10.3390/diagnostics16193172)
Supplement: Supplementary file 1 [file diagnostics-16-03172-s001.zip › diagnostics-4478191-supplementary.pdf]

Supplementary Table S1. Procedural characteristics in different types of leads

Characteristics	Total N=42	Lumen-less lead N=20	Stylet-driven lead N=22	P-value †
**Procedural characteristics**				
LBBAP procedure time (min)	32.3 ± 20.3	31.7 ± 17.5	32.8 ± 23.0	0.855
LBBAP attempts (times)	2.3 ± 1.1	2.4 ± 1.0	2.3 ± 1.2	0.927
LBBAP fluoroscopy time (sec)	69.4 ± 67.5	75.7 ± 83.2	63.6 ± 50.64	0.568
Total procedure time (min)	83.8 ± 26.3	86.4 ± 28.7	81.4 ± 24.5	0.550
Total fluoroscopy time (sec)	118.8 ± 82.1	130.5 ± 87.6	108.3 ± 77.4	0.389

Data are presented as means ± SD; LBBAP, left bundle branch area pacing

† Comparison between lumen-less pacing lead and standard stylet-driven lead.
